# Overcoming problematic growth phenotypes in organoids from patients with monogenic GI disease

**DOI:** 10.1371/journal.pone.0309072

**Published:** 2024-11-04

**Authors:** Katlynn Bugda Gwilt, Jay R. Thiagarajah

**Affiliations:** Division of Gastroenterology, Hepatology and Nutrition, Boston Children’s Hospital, Harvard Medical School, Boston, MA, United States of America; Penn State Health Milton S Hershey Medical Center, UNITED STATES OF AMERICA

## Abstract

Patient-derived organoids provide a unique model system to explore disease-causing mutations *ex vivo*. By using organoids from duodenal or colonic biopsies of pediatric patients with intestinal epithelial disorders, we can directly assay the patient cells to tailor treatment to their unique disease state. The advent of organoid technology from patients with severe intestinal disorders such as Congenital Diarrhea Enteropathies (CoDE) and Very-Early-Onset Inflammatory Bowel Disease (VEO-IBD) has allowed for rapid advances in the understanding of and the treatment of these monogenic disorders. Still, the expansion of these lines for scalable studies is not trivial, and success rates of expansion are variable between groups, and even lab members within the same group. These protocols have been validated on patients with CoDE or VEO-IBD and age-matched control patients. Here, we present our recommended protocols for the cultivation of organoids from pediatric patients with CoDE and VEO-IBD. These protocols have been validated on organoids generated from the duodenum (duodenoids), ileum (ileoids), colon (colonoids) and iPSC-derived intestinal colonoids from pediatric healthy donors or donors with CoDE or VEO-IBD (Gwilt et al., 2023). Using our modified culture media, extended culture times from biopsy preparation and thawing frozen lines, gentle passaging techniques with the incomplete removal of the organoids from the matrigel, and modified monolayer protocols (Maeda et al., 2023; Maeda et al., 2022), we have been able to successfully culture and expand several lines for more than 5 years. The conditions and protocols used here provide a basis for reproducible phenotypes, scaling for larger functional studies on patient lines, and for reproducibility of results between several investigators. We provide a useful starting point and troubleshooting guidelines for the optimization of culturing organoids from any patient with novel disease pathology.

## Introduction

Congenital Diarrhea Enteropathies (CoDE) and Very-Early-Onset Inflammatory Bowel Disease (VEO-IBD) are a group of intestinal disorders resulting from a primary epithelial cell dysfunction or altered immune cell function. Loss-of-function mutations in the gene encoding tetratricopeptide repeat domain 7A (TTC7A) cause severe infantile-onset gastrointestinal disease, characterized by profound epithelial architecture defects and overall epithelial dysfunction. Several known variants in TTC7A can lead to CoDE [1] and VEO-IBD [2, 4]. Colonoids derived from biopsies from patients with distinct point mutations in TTC7A have proven challenging to expand across multiple labs due to their susceptibility to apoptosis [1]. To address the issues encountered in the cultivation of organoids from patients with unique TTC7A mutations, we have developed protocols to facilitate the expansion of colonoids from TTC7A patients, alongside other candidate genes involved in CoDE and VEO-IBD.

Unlike existing methods, our protocol modifies the media composition to bolster stem cell proliferation, and incorporates modifications in passaging, such as retaining matrigel during the passaging and mechanical fragmentation. This methodology has demonstrated a reproducible expansion and differentiation phenotype in 3D and in 2D monolayers. Furthermore, functional studies can be conducted using these methods with dependable reproducibility across lab members. This protocol has proven useful for expanding organoids from both healthy donors as well as CoDE patients, providing material from all patients in our studies for over 5 years. Throughout this period there has been no evident reduction in growth efficiency, no alterations in differentiation of lines, and no issues with reproducibility in biochemical and functional assays.

One significant improvement over previous passaging protocols is the elimination of lengthy incubations on ice and on shakers, omission of EDTA during passaging, and the retention of the matrigel during routine passaging, which protects the cells against shear stress caused by mechanical fragmentation. On average, passaging takes about 30 minutes, with observable organoid regrowth seen within 24 hours of sub-culturing. We forego enzymatic passaging due to observed decreases in viability in several patient lines with Trypsin-EDTA passaging. For monolayer plating, we employ a brief trypsinization step combined with mechanical disruption of the cysts, adapting techniques we’ve previously described for plating monolayers [5] to successfully plate patient monolayers.

Finally, our procedures for processing fresh and frozen biopsies have achieved nearly a 100% success rate in generating organoid lines challenging patient biopsies. This protocol is an update to our previously published protocol on protocols.io (S1 File) [6]. Here, we provide greater detail into media preparations, organoid expansion, biopsy processing, and include monolayer protocols for functional assays. All outlined procedures have demonstrative supportive data.

## Materials and procedures

The protocol described in this peer-reviewed article is published on protocols.io, https://urldefense.com/v3/__https://dx.doi.org/*dx.doi.org/10.17504/protocols.io.dm6gp33ypvzp/v1*__;W10!!NZvER7FxgEiBAiR_!se0UBJZFPCgV5MGHNVEOaffMUwXcASaF2fKuliJwkqefp-l8ZucS4GWwD1dViO1YRFtLpw4wh-_ILXuodri1wdmMW8YguDox$ and is included for printing as supporting information S1 File with this article https://www.protocols.io/view/overcoming-problematic-growth-phenotypes-in-organo-cyvdxw26.

## Expected results

Using the protocol described here, we consistently expand organoids derived from patients with CoDE and VEO-IBD. An instance of this is described here. There are several unique variants in TTC7A which cause disease, with some mutations yielding a modest phenotype, and others presenting with a severe apoptotic phenotype. One such comparison can be made between two compound heterozygous patients: one with a truncating mutation–Q541x-, deleting the last 2/3 of the coding region of the transcript accompanied by a single point mutation L478P, and a second patient with a commonly seen mutation E71K and L304R.

**Fig 1:**
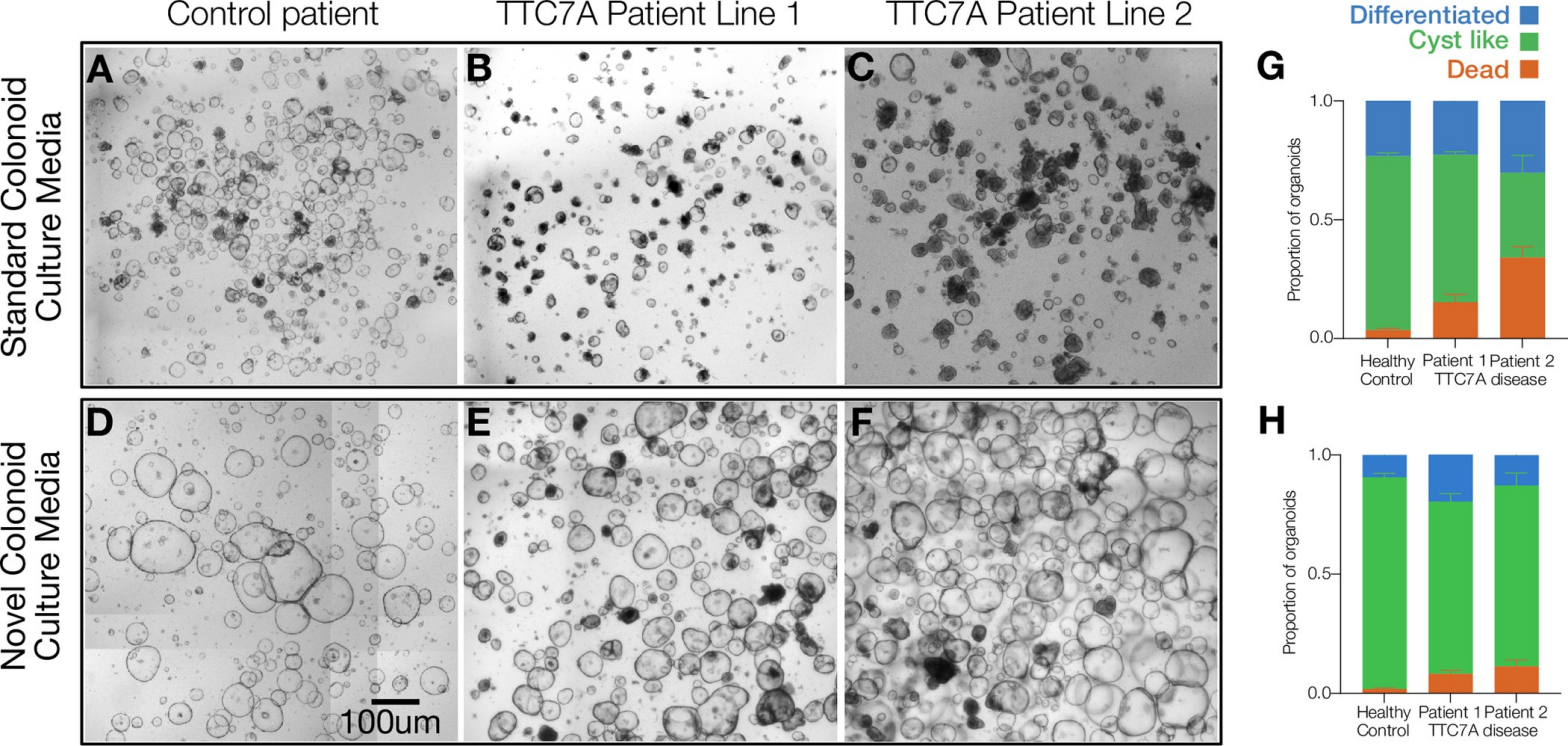
Comparison of different culture medias. A-C, Growth of Control and Patient Lines in ’Standard’ ENRW Media. D-F. Growth of control and patient lines in KBG media. G. Quantification of the number of cyst (red) like, differentiated (green) and dead (blue) colonoids grown in ’Standard’ ENRW Media. H. Quantification of the number of cyst (red) like, differentiated (green) and dead (blu) colonoid grown in supplemented media.

Initially, establishing colonoids from a patient harboring the truncating mutation posed several challenges as passage attempts with normal growth media, or dissociation from the matrigel resulted in cell death. An approach involving passaging by bringing cells to the single-cell state was trialed, but it exacerbated cell death compared to the mechanical disruption after cell recovery. Subsequently, we attempted to further modify the passaging of these lines using the protocols outlined here involving the gentle removal of 75–90% of the existing matrigel which allows organoids to maintain partial attachment to the matrix. The initial passage using these methods salvaged the patient line, though there was modest growth (Fig 1A–1C). Next, we attempted to add additional growth factors to the media as described within this protocol. Upon transitioning this patient line from the ‘standard’ culture media with 65% WRN conditioned media (CM) to media with additional Wnt/Rspondin-1/Noggin CM and increased EGF from 50ng/ml to 100ng/ml, we saw a significant increase in the viability of this line (Fig 1D–1H). Given that these procedures were so successful in resolving the limitations with this patient line, we have since transitioned several CoDE patient lines and VEO-IBD patient lines to this workflow, including our TTC7A-E71K/L304R patient, due to similar issues with viability.

**Fig 2:**
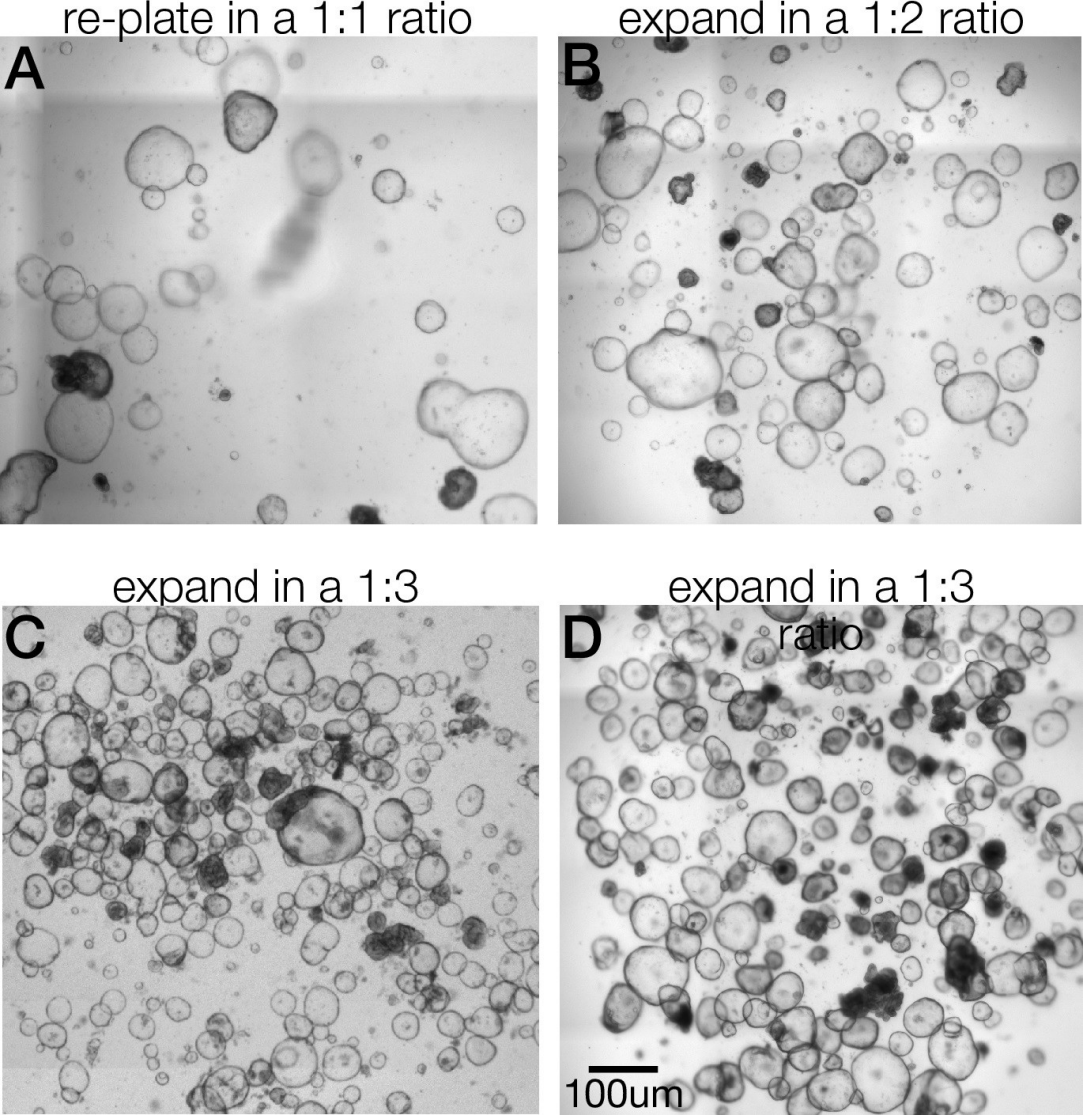
Recommended passage ratios in healthy control colonoids. A. Diffuse but large colonoids present within a 35ul matrigel drop. Fragment these organoids and replate in the same number of wells started with. B. Large colonoids, normal confluency. Fragement these organoids and expand in a 1:2 ratio. (i.e. 4 wells to 8 wells). C&D. Two examples of a 1:3 passaging ratio with highly confluent matrigel droplets. Fragment these organoids and expand in a 1:3 ratio (i.e 4 wells to 12 wells).

**Fig 3:**
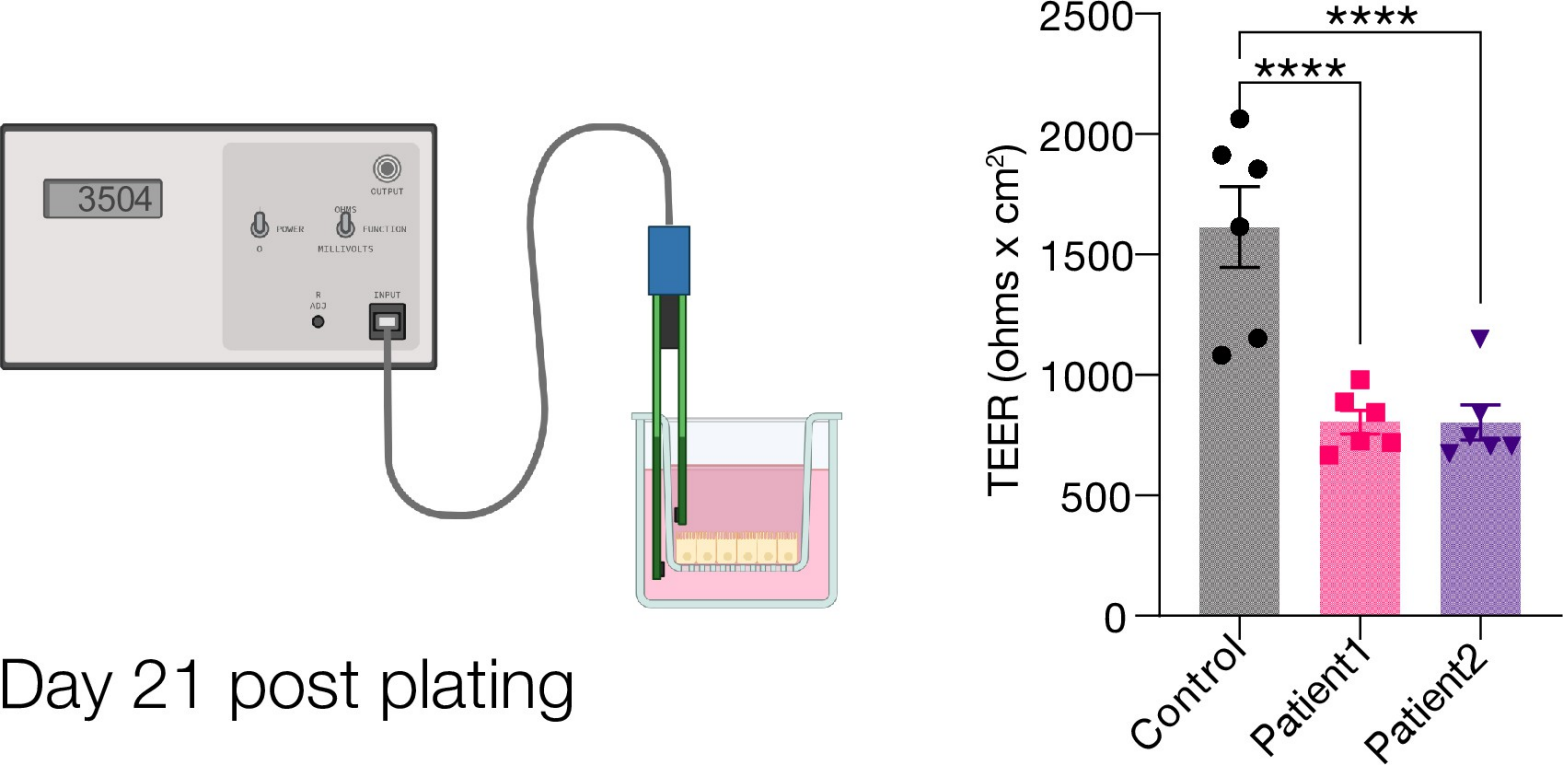
A. Schematic representation of TEER measurements. B. Representative graph of TEERs observed with control and patient lines. Data is presented as Ohms x cm^2^.

With these protocols providing a reasonably scalable growth of our patient lines, we next adapted our monolayer plating procedure to mirror this passaging protocol. Rather than using a lengthy cell recovery step followed by enzymatic disruption^2^, we modified our passaging protocol to include a very brief enzymatic digestion followed by mechanical fragmentation of lines. In doing so, we have carried out several assays on monolayers, evaluating monolayer permeability and performing functional studies to characterize barrier defects (Figs 2 and 3).

## Supporting information

S1 File (PDF)
