# Supplementary material for: Overcoming problematic growth phenotypes in organoids from patients with monogenic GI disease
Source: PLoS One. 2024 Nov 4;19(11):e0309072. doi: 10.1371/journal.pone.0309072 (PMC11534248; doi:10.1371/journal.pone.0309072)
Supplement: S1 File — (PDF) [file pone.0309072.s001.pdf]

Oct 02, 2023

Overcoming problematic growth phenotypes in organoids from patients with monogenic GI disease

DOI
dx.doi.org/10.17504/protocols.io.dm6gp33ypvzp/v1

Katlynn Bugda Gwilt[1], Jay R. Thiagarajah[1]

[1]Boston Children's Hospital

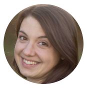

Katlynn Bugda Gwilt
Boston Children's Hospital

OPEN ACCESS

DOI: dx.doi.org/10.17504/protocols.io.dm6gp33ypvzp/v1

Protocol Citation: Katlynn Bugda Gwilt, Jay R. Thiagarajah 2023. Overcoming problematic growth phenotypes in organoids from patients with monogenic GI disease. **protocols.io** https://dx.doi.org/10.17504/protocols.io.dm6gp33ypvzp/v1

License: This is an open access protocol distributed under the terms of the **Creative Commons Attribution License**, which permits unrestricted use, distribution, and reproduction in any medium, provided the original author and source are credited

Protocol status: Working
We use this protocol and it's working

Created: August 18, 2023

Last Modified: October 02, 2023

Protocol Integer ID: 86661

Funders Acknowledgement:
NIH NIDDK
Grant ID: F32DK130267
NIH NIDDK
Grant ID: DK118640
National Institute of General Medical Sciences
Grant ID: DK118640
NIH NIDDK
Grant ID: DK034854

Abstract

Patient-derived organoids provide a unique model system to explore disease causing mutations ex vivo. By use of organoids from duodenal or colonic biopsies of pediatric patients with intestinal epithelial disorders, we can directly assay the patient cells to tailor treatment to their unique disease state. The advent of organoid technology from patients with severe intestinal disorders such as either Congenital Diarrhea Enteropathies (CoDE) and Very-Early-Onset Inflammatory Bowel Disease (VEO-IBD) has allowed for rapid advances in the understanding of and the treatment of these monogenic disorders. Still, the expansion of these lines for scalable studies is not trivial, and success rates of expansion are variable between groups, and even lab members within the same group. These protocols have been validated on patients with CoDE or VEO-IBD and age matched control patients. Here, we present our recommended protocols for the cultivation of organoids from pediatric patients with CoDE and VEO-IBD. These protocols have been validated on organoids generated from the duodenum (duodenoids), ileum (ileoids), colon (colonoids) and iPSC derived intestinal colonoids from pediatric healthy donors or donors with CoDE or VEO-IBD1. Using our modified culture media, extended culture times from biopsy preparation and thawing frozen lines, gentle passaging techniques with the incomplete removal of the organoids from the matrigel, and modified monolayer protocols2,3, we have been able to successfully culture and expand several lines for >5 years. The conditions and protocols used here provide a basis for reproducible phenotypes, scaling for larger functional studies on patient lines, and for reproducibility of results between several investigators. We provide a useful starting point and troubleshooting guidelines for optimization of culturing organoids from any patient with novel disease pathology

Materials

24-well plate polyester Transwells, 0.4 um pore size (Corning 3470)

TrypLE from LifeTechnologies cat # 1265010, aliquot and store -20C protected from the light until use.

Collagen IV, from human placenta (Sigma C5533)

Corning® Matrigel® Basement Membrane Matrix, Phenol Red-free, LDEV-free, 10 mL, Product Number:356237

Note

Ensure protein concentration of your matrigel lot is >9mg/ml prior to purchasing
Amidst supply chain issues with matrigel, we trialed matrigel containing phenol red and report no differences with our control or patient lines.

Advanced DMEM/F12: Gibco 12634-028
FBS: Normal FBS supplier for your lab
Anti/Anti: Gibco 15240062

L-WRN Conditioned Media: ATCC CRL-3276
L-Wnt3a Cell Conditioned Media: ATCC CRL-2647

293T HA-R-Spondin 1-Fc conditioned media: SigmaSCC111
293T Noggin-FC Conditioned media: Gift, Hans Clevers "
GlutaMax: Gibco 35050-061

HEPES: Gibco15630-080
Primocin: Invivogen ant-pm-2
Normocin: Invivogen ant-nr-2
B27: Gibco 12587010
N2: Gibco 17502-048
Nicotinamide: Sigma N0636

N-Acetyl-Cysteine: Sigma A8199

A8301: Sigma SML0788

SB202190: Sigma S7067

EGF: Peprotech 315-09
Gastrin: Sigma G9145

Prostaglandin E2: Sigma P5640

FGF-4: Stemcell 78103.2
Y-27632: Sigma Y0503

CHIR 99021: Stemcell 72054

Before start

Ensure that your lab has an up-to-date IRB allowing for human subject research.

Preparation of Media

1 Prepare conditioned media (CM) from L-WRN cells, L-Wnt3a cells, 293T-R-Spondin-1 Cells, and 293T-Noggin Cells as described previously.

Note

Briefly, for bulk production, expand cell lines to a large enough density to seed at least 3, T225cm^2 cell culture flasks. Prior to the production of CM, the cell lines can be reselected with antibiotic to ensure that the cell population is pure. Transition the cells to DMEM/10% FBS. Allow cells to incubate with media for 7 days, collect the culture media, centrifuge at 1000xg for 5 minutes, filter through a 20uM. Assays to validate the production of the growth factors in the media such as the TOPFLASH assay or a coomassie blot should be considered.

1.1 Aliquot L-WRN media into single use aliquots - recommended at 25ml each for duodenum expansion media or 37.5ml aliquots for colonoid expansion media.

Note

It is important to limit freeze thaw cycles of CM as well as heat-cool cycles of CM to limit growth factor degradation.

1.2 Create a Wnt3A, R-Spondin-1 and Noggin single use aliquot by combining each component into one tube as follows:

- 10 ml Noggin CM
- 5 ml Wnt3A CM
- 10 ml R-Spondin 1

2 Prepare base media as follows:

Base Media

- Advanced DMEM/F12 ~80% (v/v)
- FBS (as supplied) ~20% (v/v)
- Pen/Strep (100x)* ~1% (v/v)

3 Prepare Colonoid or Duodenoid Expansion Media as follows:

Colonoid Expansion Media

- L-WRN Conditioned Media 50% (v/v)
- Wnt/Rspondin-1/Noggin CM Mix 25% (v/v)
- Base Media 25% (v/v)
- GlutaMax 1x
- HEPES (10mM)
- Primocin 1x
- Normocin 1x

- B27 1X
- N2 1X
- Nicotinamide 10mM
- N-Acetyl-Cysteine 500µM
- A8301 500nM
- SB202190 10µM
- EGF 100ng/mL
- Gastrin 10nM
- Prostaglandin E2 100nM

Duodenoid Expansion Media

- L-WRN Conditioned Media 40% (v/v)
- Wnt/Rspondin-1/Noggin CM Mix 20% (v/v)
- Base Media 32% (v/v)
- GlutaMax 1x
- HEPES (10mM)
- Primocin 1x
- Normocin 1x
- B27 1X
- N2 1X
- Nicotinamide 10mM
- N-Acetyl-Cysteine 500µM
- A8301 500nM
- SB202190 10µM
- EGF 100ng/mL
- Gastrin 10nM

Note

These expansion media outlined here contain higher levels of Noggin R-Spondin 1, and is recommended for diseased patient lines. Healthy control human lines exhibit rapid growth in this expansion media.

Our 'standard' expansion media for colonoids consist of 65% L-WRN CM, 35% Base Media and without the Wnt/Rspondin-1/Noggin CM.
For duodenoids consists of 50% L-WRN CM, 45% Base Media also without theWnt/Rspondin-1/Noggin CM.

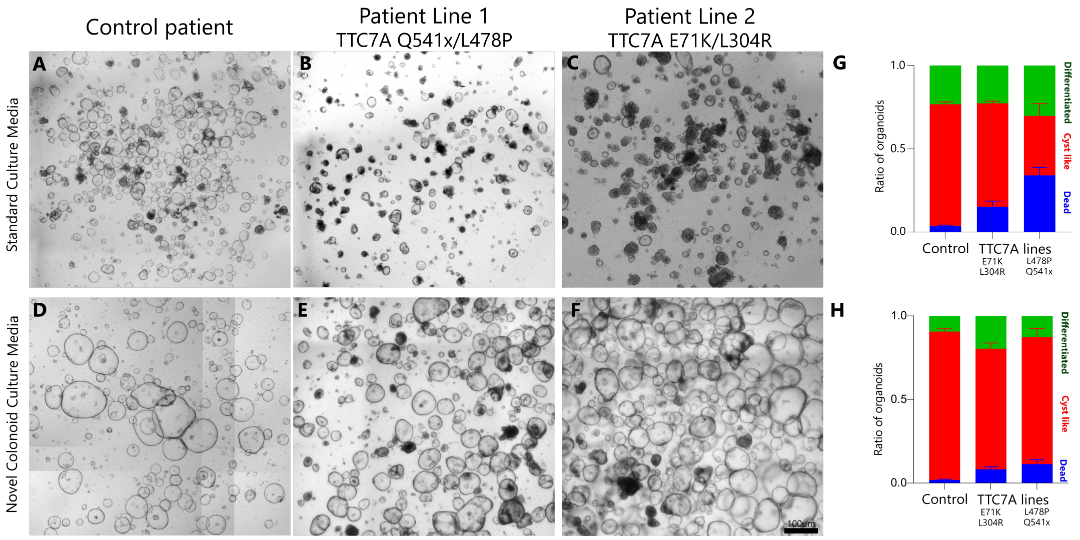

Figure 1. Comparison of different culture medias. A-C, Growth of Control and Patient Lines in 'Standard' ENRW Media. D-F. Growth of control and patient lines in KBG media. G. Quantification of the number of cyst (red) like, differentiated (green) and dead (blue) colonoids grown in 'Standard' ENRW Media. H. Quantification of the number of cyst (red) like, differentiated (green) and dead (blu) colonoid grown in supplemenented media.

4 Prepare Plating Media as follows:

Plating Media:

Add:

Y-27632 10µM & CHIR 99021 250nM

to Duodenoid or Colonoid Expansion media as prepared above

Note

We only include Y-27632 and CHIR 99021 after thawing and after splitting as outlined in subsequent sections.

5 Prepare Differentiation Media as follows:

Differentiation Media:

Base Media 90% (*v/v)*

Noggin Conditioned Media 10% (*v/v)*

Glutamax 1x

HEPES 10mM

Primocin 1x

Normocin 1x

B27 1X

A8301 500nM

EGF 50ng/ml

Gastrin 10nM

Thawing Frozen Lines

6 Thaw organoid vial in a 37C water bath until a small ice chunk remains

7 Using P1000 move frozen cells from vial to conical tube of chilled complete base media

8 300 x g Centrifuge at 300 x g for 5 minutes

9 Aspirate media leaving all of the residual matrigel from freezing behind

10 Add 200ul of fresh matrigel to the organoid/matrigel pellet

11 Gently resuspend using P1000 and 1000ul tip

12 Plate 40ul/well of a 24-well plate and allow to polymerize for 20 minutes at 37°C

13 Add 1ml of Plating Media per well

14 Keep in the 1ml Plating Media for ~72 hrs (i.e. Fri-Mon)

15 Resume every other day media changes after the initial 72 hour period

Organoid Expansion

16 Room temperature When organoids are highly stem like, add 1.0 mL of RT PBS to each well containing matrigel and organoids.

17 Gently scrape the matrigel from the bottom of the well using a P1000 tip and transfer the entire contents of the well to to a 15ml conical tube.

18 300 x g Centrifuge the organoids at 300 x g for 5 minutes.

19 Aspirate the PBS and the upper most layer of matrigel.

Note

The goal here is not to aspirate all of the matrigel. There should be some remaining on top of the organoid pellet. The goal is to remove approximately 1/2 of the old matrigel

20 Room temperature Wash the remaining pellet and matrigel once more with RT PBS and pellet organoids.

21 300 x g Centrifuge the organoids at 300 x g for 5 minutes.

22 Aspirate the PBS and remove as much of the matrigel as possible leaving only the lower-most layer of matrigel that has organoids suspended.

23 Room temperature Add 1ml of complete base medium.

24 Using P200 pipet and pipette tip, triturate at medium intensity. Triturate approximately 30 times. Organoids should be fluffy floating in the media along the edges. For diseased lines, pipette with less force to retain viability.

25 On ice Add 4 ml of ice-cold PBS to the fragmented organoids to facilitate the removal of debris.

26 300 x g Centrifuge the organoids at 300 x g for 5 minutes.

27 Aspirate JUST the PBS/base media mixture leaving almost no media on top of the matrigel pellet

28 Add 50ul of full growth media to the pellet and resuspend

29 Add 35ul of fresh matrigel per expected wells to the pellet and resuspend slowlyto avoid bubbles.

Note

The amount of matrigel added is directly proportional to the confluency of your organoids being passaged, see below figure.

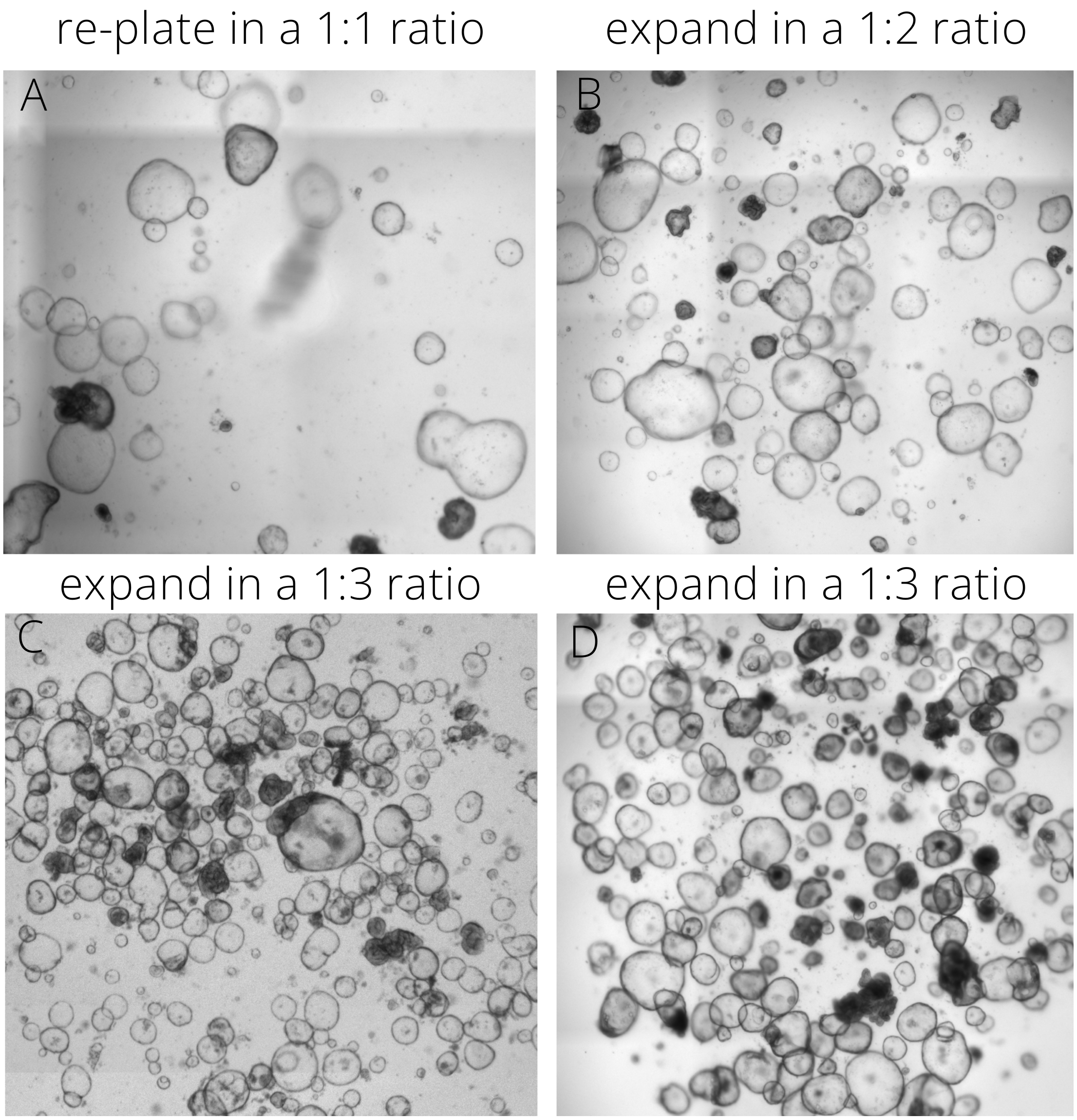

Figure 2. Recommended passage ratios in healthy control colonoids. A. Diffuse but large colonoids present within a 35ul matrigel drop. Fragment these organoids and replate in the same number of wells started with. B. Large colonoids, normal confluency. Fragement these organoids and expand in a 1:2 ratio. (i.e. 4 wells to 8 wells). C&D. Two examples of a 1:3 passaging ratio with highly confluent matrigel droplets. Fragment these organoids and expand in a 1:3 ratio (i.e 4 wells to 12 wells).

30 Plate matrigel and organoids in 35ul droplets in the center of the well of a 24-well plate.

31 Add 500 ul of media. Continue with media changes every other day until experiment.

Plating Patient-derived organoids as monolayers

32 Expand organoids to plate in a ratio of 1.5 wells/2 transwells (Figure 2B) or 1.5 well/2 transwells (Figure 2C/D). Ensure that the organoids are still cyst like with little differentiation.

33 Prepare stock collagen by dissolving to a final concentration of 1mg/ml in 100 mM acetic acid. Aliquot and store at -20C until use.

34 Prepare working concentration of collagen by dissolving the stock collagen to a working concentration of 33ug/ml collagen in cell culture water

35 To coat each transwell well with collagen, add 100ul of working concentration (33ug/ml) of collagen to each transwell. Be sure the entire area of the transwell is coated with collagen.

36 Overnight Be sure the plate is covered and incubate at 4°C overnight.

37 On the day of plating wash both the apical and basolateral chamber with base media prior to plating organoids.

38 Aspirate medium from each well of the 24 plate and add 1.0 mL of **cell recovery/dissociation solution** directly to the matrigel.

39 Scrape the matrigel from the bottom of the plate and transfer the entire contents of the well to a 15ml conical tube.

40 300 x g Centrifuge the organoids at 300 x g for 5 minutes.

41 Aspirate the cell recovery solution and wash the remaining pellet once more with ice cold cell recovery media.

42 300 x g Centrifuge the organoids at 300 x g for 5 minutes.

43 Aspirate the cell recovery solution and remove as much of the supernatant as possible

44 Add undiluted Trypsin+EDTA to enteroids; mix gently 5X with P200.

45 37 °C Place in a 37C water bath for ***absolutely no greater than*** 90seconds.

46 Spray with ethanol prior to bringing back into the hood.

47 Immediately add 5ml of chilled complete base media.

48 Triturate with P200 to finish the fragmenting of the organoids.

49 300 x g Centrifuge the organoids at 300 x g for 5 minutes.

50 Aspirate the supernatant and gently suspend the organoid fragments in the plating media using a P200. Add 160ul for every transwell to be plated.

51 After the transwells are washed, add 1000µl of plating media to the basolateral chamber.

52 Make sure the organoid pellet is evenly dispersed when resuspended. Pipet 155µl of organoids to the center of each transwell SLOWLY.

53 GENTLY place the plate in the incubator. We recommend placing at the back of your incubator shelf at 37°C.

Expected result

For the first 3-4 days, the monolayer may look as if cells have all died if looked at under the microscope. This is normal. A small percentage of the organoids will die. This is why the initial plating density is so high. For this reason, we advise waiting until day 4 post plating to change media and image growing monolayer.

54 On day 4 post plating, gently bring the plate out of the incubator and into the TC hood, and aspirate the basolateral media without disturbing the apical media. Add 1000µl of fresh media to each transwell.

55 **Do not change the apical media until day 6 post plating.**

56 Proceed with every other day media changes as your monolayer becomes stable. The monolayer will take 7-14 days to become confluent.

57 Once the monolayer is fully confluent, switch to differentiation media for 5 days.

58 On day 5, post-differentiation media, take the transepithelial electrical resistance (TEER) of the monolayer.

Note

To measure TEER, sterilize the electrode prong with 70% ethanol and plug in the probe on the EVOM. Equilibrate the probe in a falcon tube with differentiation media for 5 minutes. Once the EVOM is consistently reading zero, gently place the short prong in the apical reservoir and the long prong in the basolateral reservoir. Once the reading on the EVOM steadies, record the TEER. We do not recommend checking the TEER daily as there is a decrease in viability of the monolayer due to the electrical current.

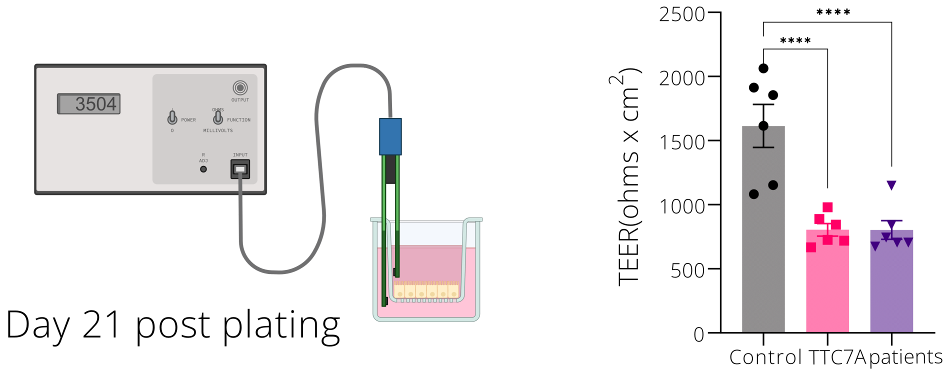

Figure 3. A. Schematic representation of TEER measurements. B. Representative graph of TEERs observed with control and patient lines. Data is presented as Ohms x cm^2 .

59 Proceed with functional experiment.

Protocol references

1. Gwilt,
K. B. & Thiagarajah, J. R. Strategies for optimizing the isolation and
expansion of sensitive patient-derived duodenoids, ileoids and ... (2023).

2. Maeda, K., Bugda Gwilt, K., Schmieder,
S. S., Zachos, N. C. & Lencer, W. I. Protocol for measuring transcytosis
and recycling of IgG in intestinal epithelial Caco-2 cells and primary human
intestinal organoids. *STAR Protoc.* **4**, 102335 (2023).

3. Maeda, K. *et al.* Depletion of the
apical endosome in response to viruses and bacterial toxins provides
cell-autonomous host defense at mucosal surfaces. *Cell Host Microbe* **30**,
216-231.e5 (2022).

4. Jardine, S. *et al.* Drug Screen
Identifies Leflunomide for Treatment of Inflammatory Bowel Diseases Caused by
TTC7A Deficiency. *Gastroenterology* **158**, 1000–1015 (2020).

5. Avitzur, Y. *et al.* Mutations in
tetratricopeptide repeat domain 7A result in a severe form of very early onset
inflammatory bowel disease. *Gastroenterology* **146**, 1028–1039
(2014).

6. Dhingani, N. *et al.* The E3
ubiquitin ligase UBR5 interacts with TTC7A and may be associated with very
early onset inflammatory bowel disease. *Sci. Rep.* **10**, 18648
(2020).

7. Sato, T. *et al.* Single Lgr5 stem
cells build crypt-villus structures in vitro without a mesenchymal niche. *Nature* **459**, 262–265 (2009).

8. Fujii, M. *et al.* Human Intestinal Organoids Maintain Self-Renewal Capacity and Cellular Diversity in Niche-Inspired Culture Condition. *Cell Stem Cell* **23**, 787-793.e6 (2018).

9. VanDussen, K. L., Sonnek, N. M. & Stappenbeck, T. S. L-WRN conditioned medium for gastrointestinal epithelial stem cell culture shows replicable batch-to-batch activity levels across multiple research teams. *Stem Cell Res.* **37**, 101430 (2019).
